# L-lactic acid production by *Aspergillus brasiliensis* overexpressing the heterologous *ldha* gene from *Rhizopus oryzae*

**DOI:** 10.1186/s12934-015-0249-x

**Published:** 2015-05-03

**Authors:** Nadège Liaud, Marie-Noëlle Rosso, Nicolas Fabre, Sylvaine Crapart, Isabelle Herpoël-Gimbert, Jean-Claude Sigoillot, Sana Raouche, Anthony Levasseur

**Affiliations:** INRA, UMR1163 Biodiversité et Biotechnologie Fongiques, Polytech’ Marseille, 163 avenue de Luminy, CP 925, 13288, Marseille, Cedex 09 France; Aix-Marseille Université, UMR1163 Biodiversité et Biotechnologie Fongiques, Polytech’ Marseille, 163 avenue de Luminy, CP 925, 13288, Marseille, Cedex 09 France; ARD Agro-Industrie Recherche et Développement, Route de Bazancourt, 51110, Pomacle, France; Aix-Marseille Université, Unité de Recherche sur les Maladies Infectieuses et Tropicales Emergentes (URMITE), UM63, CNRS 7278, IRD 198, INSERM U1095, IHU Méditerranée Infection, Pôle des Maladies Infectieuses, Assistance Publique-Hôpitaux de Marseille, Faculté de Médecine, 27 Bd Jean Moulin, 13005 Marseille, France

## Abstract

**Background:**

Lactic acid is the building block of poly-lactic acid (PLA), a biopolymer that could be set to replace petroleum-based plastics. To make lactic acid production cost-effective, the production process should be carried out at low pH, in low-nutrient media, and with a low-cost carbon source. Yeasts have been engineered to produce high levels of lactic acid at low pH from glucose but not from carbohydrate polymers (e.g. cellulose, hemicellulose, starch). *Aspergilli* are versatile microbial cell factories able to naturally produce large amounts of organic acids at low pH and to metabolize cheap abundant carbon sources such as plant biomass. However, they have never been used for lactic acid production.

**Results:**

To investigate the feasibility of lactic acid production with *Aspergillus*, the NAD-dependent lactate dehydrogenase (LDH) responsible for lactic acid production by *Rhizopus oryzae* was produced in *Aspergillus brasiliensis* BRFM103. Among transformants, the best lactic acid producer, *A. brasiliensis* BRFM1877, integrated 6 *ldhA* gene copies, and intracellular LDH activity was 9.2 × 10^−2^ U/mg. At a final pH of 1.6, lactic acid titer reached 13.1 g/L (conversion yield: 26%, w/w) at 138 h in glucose-ammonium medium. This extreme pH drop was subsequently prevented by switching nitrogen source from ammonium sulfate to Na-nitrate, leading to a final pH of 3 and a lactic acid titer of 17.7 g/L (conversion yield: 47%, w/w) at 90 h of culture. Final titer was further improved to 32.2 g/L of lactic acid (conversion yield: 44%, w/w) by adding 20 g/L glucose to the culture medium at 96 h. This strain was ultimately able to produce lactic acid from xylose, arabinose, starch and xylan.

**Conclusion:**

We obtained the first *Aspergillus* strains able to produce large amounts of lactic acid by inserting recombinant *ldhA* genes from *R. oryzae* into a wild-type *A. brasiliensis* strain. pH regulation failed to significantly increase lactic acid production, but switching nitrogen source and changing culture feed enabled a 1.8-fold increase in conversion yields. The strain produced lactic acid from plant biomass. Our findings make *A. brasiliensis* a strong contender microorganism for low-pH acid production from various complex substrates, especially hemicellulose.

## Background

Lactic acid (LA) is a colorless, odorless monocarboxylic acid naturally produced by many organisms. This weak acid has low buffering power and is tasteless, and has thus found broad use as an excipient in the food, cosmetics, pharmaceutical and chemical industries [1,2]. LA is listed in the GRAS inventory, the L-isomer being preferred for food and pharmaceutical applications due to the prevalence of the L-LA found in mammals [3]. Estimates from 2008 claim the food industry absorbs up 85% of LA production [1]. Besides its application as an additive or excipient, LA is the building block for poly-lactic acid (PLA), a biodegradable polymer that could be set to replace petroleum-based plastics. With mounting environmental pressure for sustainable industry, demand for PLA biomaterial is increasing demand for LA, for which the market is expected to hit 1 million tons by 2020 [4]. Nevertheless, the selling price of PLA still has to be cut by at least 50% to compete with fossil fuel-based plastics [2].

Industrial LA production bioprocesses traditionally use lactic acid bacteria (LAB), mainly from *Lactobacillus* species. LA is a natural end-product of carbohydrate metabolism and is produced from pyruvate with an NAD-dependent lactic acid dehydrogenase (LDH). LAB naturally produce high amounts of LA by fermentation, and yields can approach the theoretical maximum of 100% (w/w) for homolactic fermentation in complex media [1,3,5,6]. However, LA production by LAB is expensive due to their complex nutrient requirements and the restrictive use of pH neutralizers (e.g. CaCO_3_) during fermentation [5]. To decrease LA production costs and expand the market for LA and its green derivatives, low-cost media containing cheap carbon sources and low-pH production processes are needed [2]. Low-pH production processes that do not require neutralizing chemicals allow undissociated (free) LA to be recovered directly from the broth without any acidification step in the purification process [1].

Members of the fungal kingdom known to have low nutrient requirements and to be acid-tolerant are considered an attractive solution for low-pH LA production. Research into genetically-modified yeasts expressing heterologous *ldh* genes has intensified, and efficient low-pH production of LA has been obtained in yeasts from *Saccharomyces* [7-11], *Kluyveromyces* [12] and *Candida* genera [13-15]. This engineered yeast-based strategy has reached industrial application, and L-LA is currently produced from fermentable sugars released from pretreated corn starch, sugarcane or sugar beet [2].

Direct conversion of starch or cheaper carbon sources such as raw plant biomass would further reduce the costs of L-LA production. Using lignocellulose as carbon source entails enzymatic depolymerization of cellulose and hemicelluloses followed by assimilation of the released monomers, mainly glucose, xylose and associated arabinose [16]. *S. cerevisiae* does not naturally assimilate pentose sugars [17]. However, *Pichia stipitis* and *Candida sonorensis* are able to naturally utilize xylose, and both strains were successfully engineered for L-LA production from xylose [15,18]. Yeasts do not feature among natural lignocellulose degraders due to their poor (hemi)cellulolytic enzyme arsenal. Therefore, yeast utilization requires complex metabolic engineering before this objective is achievable [19]. In contrast, wood-degrading and saprophytic fungi such as filamentous fungi can grow on various carbon sources and are able to produce the enzymes necessary for biomass breakdown [20].

The sole filamentous fungus known to produce large amounts of L-LA from various carbohydrate materials is *Rhizopus oryzae*, which belongs to the order Mucorales [21,22]. This fungus is naturally able to produce L-LA from glucose with yields reaching about 80% (w/w) in minimum medium [23-25]. Attempts to produce L-LA from xylose [26,27] and low-cost carbon sources such as raw starch [25], wheat straw [28] and cellulose [29] have already been successfully performed [23], but L-LA production using *Rhizopus* still requires near-neutral pH conditions, which heavily compromises the global process [21-24]. To our knowledge, no other filamentous fungus has been studied for L-LA production. *Aspergillus* is a very powerful microbial cell factory that has been used for decades to produce organic acids such as citric acid (*A. niger*) and itaconic acid (*A. itaconicus* and *A. terreus*) at low pH and at industrial scales [24]. This low-pH organic acid production suggests that *Aspergilli* are able to endure the weak acid stress caused by organic acid accumulation, which makes them promising candidates for low-pH L-LA production. Furthermore, *Aspergilli* secrete diverse hydrolytic and oxidative enzymes involved in the breakdown of complex carbohydrates [30] and are able to metabolize pentoses from plant biomass degradation. This ability could enable L-LA production from plant biomass, thus cutting down the cost of carbon source.

The aim of this study was to investigate whether *Aspergillus brasiliensis* could be an efficient alternative to yeast for L-LA production at low pH. *A. brasiliensis*, a species from the *Nigri* section of *Aspergillus* [31], has been suggested to be safer for industrial utilization than *A. niger* since it is unable to produce fuminosin and ochratoxins, which are carcinogenic mycotoxins [32]. In particular, *A. brasiliensis* strain BRFM103 is able to accumulate more ethanol than strains from other *Aspergillus* species in minimum medium without pH regulation [33]. Both lactic acid and ethanol are produced from pyruvate, in the cytosol, with the regeneration of NAD^+^ cofactors. Lactic acid fermentation will provide an alternative route for the regeneration of NAD^+^, potentially replacing ethanol production. L-LA-producing strains of *A. brasiliensis* were obtained by multicopy integration of the heterologous *ldhA* gene from *R. oryzae*. Gene copy number and subsequent LDH intracellular activity were determined in order to study their impact on L-LA production yields. The impact of pH on L-LA production was investigated with the strain showing the highest intracellular LDH activity. Finally, L-LA production was tested with various carbohydrate substrates.

## Results and Discussion

### Selection of transformants in ammonium medium without pH regulation

**Figure 1:**
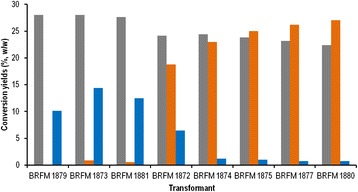
Conversion yields of glucose to biomass, lactic acid, and ethanol by the L-LA-producing transformants of *A. brasiliensis*. Conversion yields of lactic acid at 138 h of culture (orange square), ethanol at 138 h of culture (blue square), and dry biomass at 144 h of culture (gray square), are expressed in g/100 g of glucose consumed. Transformant strains are sorted on the basis of their L-LA conversion yields.

After co-transformation of *A. brasiliensis* BRFM103 with the lactate dehydrogenase A gene (*ldhA*) and the hygromycin resistance gene (*hph+*), isolated transformants were screened for L-LA production in MM1 liquid medium without pH regulation. pH of the medium dropped from 5 to 2.5 in 40 h then slowly decreased to a final pH of 1.6 at 138 h culture. Eight transformants produced L-LA and were selected for further analysis. The supernatants of these clones were analyzed by chromatography to determine residual glucose, L-LA and by-product concentrations. At 138 h incubation, all the glucose was consumed. L-LA production at 138 h incubation was low (0.03 to 0.6 g/L) for three of the L-LA-producing transformants (BRFM1879, BRFM1873, and BRFM1881). Five recombinant strains (BRFM1872, BRFM1874, BRFM1875, BRFM1877 and BRFM1880) demonstrated high L-LA production (11.7 to 13.9 g/L) from 50 g/L of glucose at 138 h of culture. L-LA conversion yields ranged from 0.1% (w/w) for BRFM1879 to 27% (w/w) for BRFM1880 (Figure 1). Recombinant strains produced low ethanol conversion yields ranging from 1 to 14% (w/w). For the transformants exhibiting L-LA conversion yields higher than 23% (w/w), ethanol production was nearly abolished with final conversion yields below 1% (w/w), suggesting that the heterologous *ldhA* efficiently compete with native pyruvate decarboxylase (PDC) for pyruvate utilization in high L-LA-producing strains. Similar results were obtained in yeasts with native ethanol production such as *Candida sonorensis,* where the natural production of around 20 g/L of ethanol was nearly abolished by *ldh* insertion in neutralized culture conditions [13]. In *S. cerevisiae*, which naturally produces larger amounts of ethanol, additional modifications of the ethanol pathway are necessary to abolish ethanol production [34,35]. L-LA production had no severe impact on growth of the recombinant strains. However, the biomass conversion yields of high L-LA-producing strains, at 22 to 24% (w/w), were lower than the biomass conversion yields of low L-LA-producing strains, at 28 to 31% (w/w) (Figure 1).

### Gene copy number and intracellular LDH activity in recombinant strains

*ldhA* gene copy number and intracellular NADH dehydrogenase activities were determined for the recombinant *A. brasiliensis-ldhA* strains. Fresh cultures of *A. brasiliensis-ldhA* transformants in MM2 liquid medium without pH regulation were used for these experiments. The wild-type BRFM103 strain was studied as a control.

**Figure 2:**
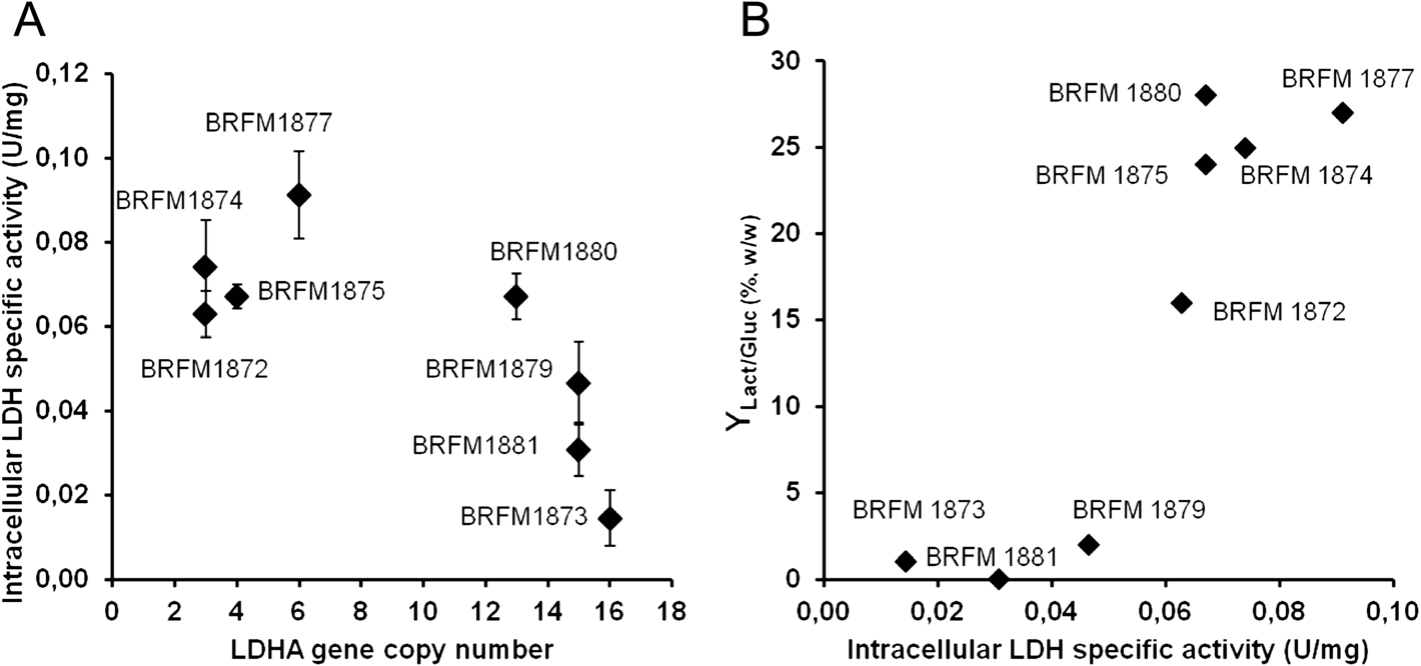
Relation between intracellular LDH activity and gene copy number **(A)** or lactic acid conversion yields **(B)**. The gene copy number was determined with fresh 24 h cultures. Activities are expressed in U per mg of total proteins, where 1 U is the amount of μmole NADH, H^+^ reduced by intracellular extract per min. For intracellular LDH activity, measurements were carried out in triplicate at 72 h of culture; error bars show the standard deviation. Conversion yields were measured during the screening assays at 138 h incubation.

The genome of LA-producing strains contained between 3 and 16 *ldhA* gene copies (Figure 2A). Intracellular LDH activities increased with *ldhA* gene copy number up to 6 copies, corresponding to a maximum LDH activity of 9.1x10^−2^ U/mg (Figure 2A). Above 6 *ldhA* gene copies, LDH activity decreased gradually to a minimal value of 1.4x10^−2^ U/mg. The reduced LDH activity could be a consequence of *ldhA* mRNA degradation due to gene silencing occurring in *Aspergillus* [36]. Furthermore, some transformants with equal gene copy numbers showed variable LDH activity levels, probably as a result of ectopic integration of the *ldhA* gene, which is a feature of the transformation system [37]. Indeed, expression level can be different in different regions of the genome.

Increasing LDH activity had a positive impact on LA yields up to 6.7x10^−2^ U/mg (Figure 2B). Above this threshold, there was no further observable increase in yield. This suggests that the LDH activity is not a limiting step in lactic acid production above 6.7x10^−2^ U/mg, and so another parameter, such as medium composition or LA toxicity, might be limiting LA production. Since high LDH activity is likely to empower LDH in the competition for pyruvate utilization, *A. niger* BRFM1877, showing the highest intracellular LDH activity, was chosen for further investigation.

### Impact of pH on L-LA production by *A. brasiliensis* BRFM1877

Over the course of liquid culture of BRFM1877 in MM1, pH of the medium dropped from 5 to 2.4 in 40 h then slowly decreased to a final value of 1.6 at 138 h of culture. This intense acidification illustrates the acid tolerance of *Aspergilli.* For citric acid production, a pH of 2 increases production yield [38]. However, at low external pH, more energy is needed for the export of lactate, since undissociated L-LA from the broth can freely reenter the cell. This is suggested to be the main reason for weak acid stress in yeasts [11,13]. In order to evaluate the impact of pH acidification during L-LA production by BRFM1877, the strain was cultured in MM2 liquid medium (50 g/L glucose initial) containing Na-nitrate as nitrogen source instead of ammonium sulfate. In this experiment, extra glucose (20 g/L) was added at 96 h growth to increase final titers.

**Figure 3:**
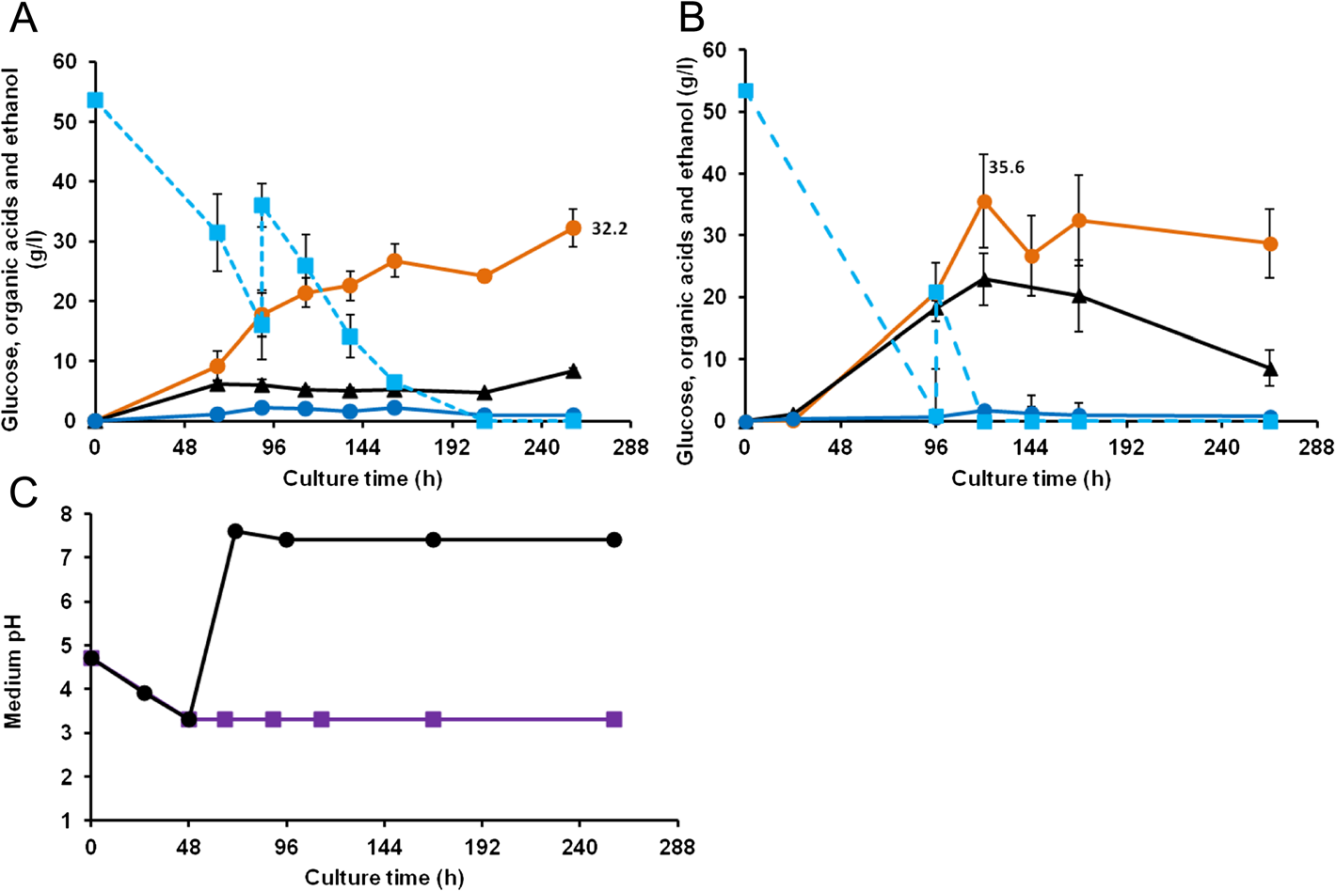
Impact of pH on organic acid production by *A. brasiliensis* BRFM1877 in glucose–nitrate-containing medium. Glucose (blue square), gluconic acid (black triangle), ethanol (blue circle), and lactic acid (orange circle) concentrations in cultures carried out in MM2 liquid medium started with 50 g/L of glucose then added with 20 g/L of glucose at 96 h of culture, without **(A)** and with **(B)** addition of 80 g/L CaCO_3_ after 24 h of culture. The evolution of pH in both cultures, with (black triangle) and without (violet square) CaCO3 addition, is also presented **(C)**. Experiments were carried out in triplicate. Error bars show standard deviations.

With Na-nitrate as nitrogen source, pH dropped from 5 to 3.2 in 48 hours then remained unchanged during further culture. The L-LA titers and conversion yields reached mean values of 17.7 g/L and 32.2 g/L, 47% (w/w) and 44% (w/w), at 90 h (before extra glucose addition) and 260 h of culture, respectively (Figure 3A). These titers and yields are higher than those obtained at pH below 2 (13.1 g/L, 26%, w/w), highlighting that ammonium sulfate as a nitrogen source restricted L-LA production, most probably via strong pH acidification. Na-nitrate was therefore used as nitrogen source to further investigate the impact of pH on L-LA production by *A. brasiliensis* BRFM1877.

Cultures at near-neutral pH were performed in MM2 liquid medium with CaCO_3_ addition and extra glucose addition at 96 h. CaCO_3_ was added after spore germination (24 h) at a final concentration of 80 g/L. Immediately after CaCO_3_ addition, pH increased from 4.0 to 6.5 and stabilized around 7.5 within a few hours and throughout subsequent fungal growth. L-LA productivity was strongly improved from 0.13 g/(L.h) to 0.32 g/(L.h) by pH neutralization (Figure 3). Furthermore, the L-LA titers reached mean values of 20.8 g/L and 35.6 g/L at 96 h (before extra glucose addition) and 120 h of culture, respectively (Figure 3B). The conversion yields from 70 g/L of glucose were therefore 51% (w/w) and 46% (w/w) with and without CaCO_3_ addition, respectively. This result shows that adding CaCO_3_ has a small effect on L-LA titers and conversion yields of *A. brasiliensis* BRFM1877 in the conditions tested. Moreover, cultures at pH above the pKa of lactic acid (3.8) led to the production of calcium lactate and complicate the recovery of free lactic acid [2]. Therefore, concentration of free lactic acid will be higher in culture at pH 3 than culture at pH 7.5 (with CaCO_3_ addition).

The low effect of pH neutralization on L-LA titers and conversion yields could be explained by the significant increase of gluconic acid. The titer of gluconic acid obtained in neutralized conditions was 22.9 g/L whereas only 6.2 g/L was produced at pH 3. Glucose oxidase, the enzyme responsible for gluconic acid production, is known to be less efficient at a pH below 3 [38]. Therefore, the slight positive effect of pH neutralization on lactic acid production might be attenuated by the negative effect of gluconic acid production. One additional advantage of culturing *A. brasiliensis* BRFM1877 at pH 3 is that it reduces the production of gluconic acid without genetic modification of the gluconic acid metabolic pathway. Studies on recombinant yeast strains expressing heterologous *ldh* genes have reported various effects of pH on L-LA production [7,13,39,40], the most prevalent being the effect of host genetic background and origin of the heterologous LDHA on low pH L-LA production [13,41]. Looking at the recombinant yeasts expressing the same *ldh* gene from *R. oryzae*, *A. brasiliensis* BRFM1877 compares favorably for low-pH L-LA production. For instance, the production yield of *S. cerevisiae* cultivated at pH 3.5 was 32% (29.2 g/L of L-LA from 92 g/L of glucose) [40] whereas *C. sonorensis* cultivated without pH regulation produced 12% of L-LA (6 g/L from 50 g/L of glucose) [13]. Here, L-LA yielded 44% (32.2 g/L of L-LA from 70 g/L of glucose) in nitrate minimum medium at pH 3, demonstrating that *A. brasiliensis* BRFM1877 shows promising performances for L-LA production at low pH.

### Production of lactic acid from various substrates by *A. brasiliensis* BRFM1877

**Figure 4:**
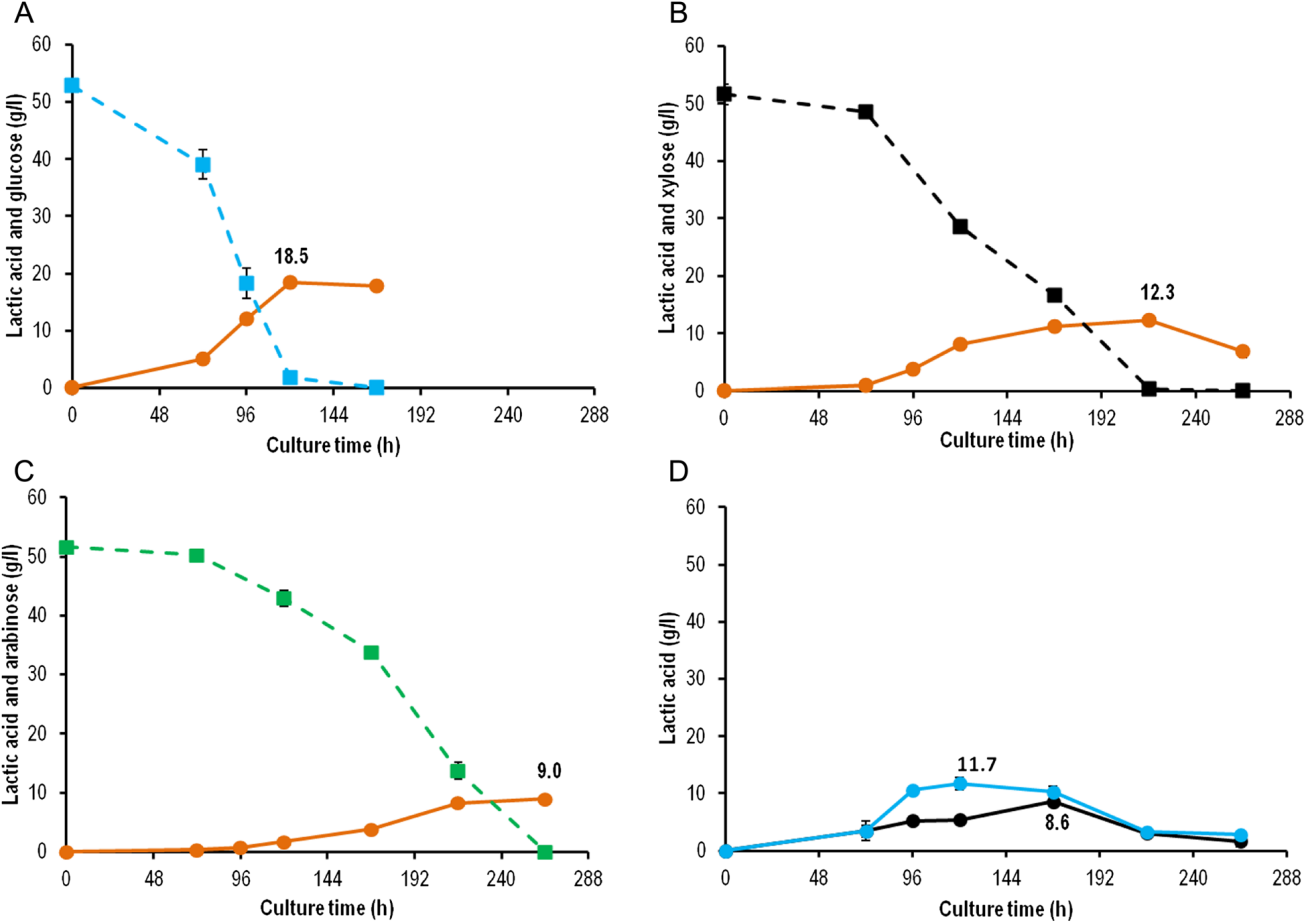
Lactic acid production and sugar consumption from monosaccharides and plant biomass polysaccharides. Lactic acid (orange square), D-glucose (blue square), D-xylose (black square), L-arabinose (green square) concentrations were determined when *A. brasiliensis* was grown on monosaccharides (A, B, C) with D-glucose **(A)**, D-xylose **(B)** or L-arabinose **(C)**. *A. brasiliensis* was also grown on polysaccharides **(D)** to produce lactic acid from starch (blue circle) or xylan (black circle). For each, initial substrate concentration was of 50 g/l and 50 g/kg for monosaccharides and polysaccharides, respectively. Experiments were carried out in triplicate. Error bars show standard deviations.

In agreement with the versatile ability of *Aspergilli* to break down the lignocellulose building blocks, we evaluated the ability of *A. brasiliensis* BRFM1877 to assimilate the main pentose monomers from hemicelluloses, *i.e.* D-xylose and associated L-arabinose, compared to the main hexose, D-glucose, in MM2 liquid medium without pH neutralization. As expected from the literature [15], the best L-LA production yield (34%, w/w) was obtained from D-glucose (Figure 4A). D-xylose was also an efficient carbon source, at 24% (w/w) L-LA production yield (Figure 4B). Using L-arabinose, production yield was 18% w/w (Figure 4C). A similar decrease in product formation when pentoses are used instead of glucose is reported for ethanol production by yeasts. [42]. A redox imbalance due to the need for NADPH in the reduction step of xylose degradation is proposed to be responsible for this decreased production [42]. In *Aspergillus,* D-xylose and L-arabinose degradation also use NADPH. Moreover, L-arabinose has a greater need for NADPH than D-xylose [43]. Similar redox imbalance could explain the lower L-LA yield observed with L-arabinose compared to D-xylose. The maximum concentration of L-LA (18.5 g/L) was obtained at 120 h culture from D-glucose (Figure 4A). Production time was extended by using pentoses. The strain produced maximal titers of 12.3 g/L L-LA at 216 h from D-xylose (Figure 4B) and 9.0 g/L at 264 h from L-arabinose (Figure 4C). The various production timescales could be explained by the lag phase due to adaptation of the strain transferred from Sabouraud broth (glucose) to media containing pentoses as carbon sources. Alternatively, fungal growth could be slowed down by its xylose and arabinose uptake rates [17].

Since *A. brasiliensis* BRFM1877 was able to produce L-LA from glucose and xylose, we investigated its ability to directly convert glucose and xylose-based polymers, *i.e.* potato starch and birchwood xylan. L-LA titers reached 11.7 g/L at 120 h and 8.6 g/L at 168 h of cultivation from starch and xylan, respectively (Figure 4D). Production yields were 23% (w/w) from starch and 17% (w/w) from xylan. The loss of yield on these polymers compared to the production yields of their corresponding monomers (glucose 34%, w/w; xylose 24%, w/w) could be attributed to the extra energy needed for the production of starch or xylan-degrading enzymes. Alternatively, L-LA production could be impacted by an incomplete degradation of the polymers. Interestingly, there was no significant lag in L-LA production times on polymers compared with their corresponding monomers. Indeed, the maximum L-LA titers were obtained at 120 h from glucose- and starch-containing media. Surprisingly, our results even suggest that xylan was consumed faster than xylose. Note that we used the same preculture to inoculate all the flasks in this experiment. This result suggests that degradation of the polymer was not a limiting step for L-LA production from starch and xylan.

The major bottleneck for cost-efficient metabolite production from polymers is the cost of enzymatic pretreatment of raw materials [23]. Here, the production of L-LA directly from starch and xylan, without addition of enzyme cocktail, emerges as a novel and promising biotechnological method for L-LA production.

## Conclusion

The overexpression of a heterologous *ldhA* gene from *R. oryzae* made it possible to produce significant amounts of L-LA by *A. brasiliensis* recombinant strains. The best L-LA producer strain, *A. brasiliensis* BRFM1877, integrated 6 *ldhA* gene copies in its genome and demonstrated an intracellular LDH activity of 9.2 x 10^−2^ U/mg. Switching the nitrogen source from ammonium sulfate to Na-nitrate prevented a strong drop in pH and increased the L-LA conversion yields. With nitrate, at pH 3, L-LA conversion yield reached 44% (w/w) with low quantities of by-product. L-LA productivity was higher at near-neutral pH than at pH 3, but the lower pH prevents the production of gluconic acid resulting in similar L-LA conversion yields with or without pH neutralization. The recombinant strain was able to produce L-LA from xylose and associated arabinose, the main pentose monomers found in plant biomass. It was also able to produce L-LA directly from starch and birchwood xylan without addition of polysaccharide depolymerizing enzyme cocktails. Further studies including process and metabolic engineering are expected to increase both titer and yield. These findings raise new prospects for using *Aspergillus* species as new hosts for L-LA production from plant biomass.

## Material and methods

### Microbial strains

*Escherichia coli* JM109 (Promega, Charbonnière, France) was used as a host for the cloning and propagation of vectors. A wild-type *A. brasiliensis* strain (BRFM103, CIRM-CF, Marseille, France) collected in a temperate forest was used as host for fungal transformation [33]. The eight recombinant L-LA-producing strains were registered in the CIRM as BRFM1872, BRFM1873, BRFM1874, BRFM1875, BRFM1877, BRFM1879, BRFM1880, BRFM1881.

### Media and culture conditions

*A. brasiliensis* strains were maintained on agar solidified minimal medium (MM) containing 10 g/L D-glucose, 70 mM NaNO_3_, 7 mM KCl, 11 mM KH_2_PO_4_, 2 mM MgSO_4_, agar 16 g/L and trace elements (76 μM ZnSO_4_, 178 μM H_3_BO_3_, 25 μM MnCl_2_, 18 μM FeSO_4_, 7.1 μM CoCl_2_, 6.4 μM CuSO_4_, 6.2 μM Na_2_MoO_4_, 174 μM EDTA-Na_2_). All medium components were from Prolabo except the Na-nitrate which was purchased from by Sigma-Aldrich.

After transformation of the parental strain, the transformants were grown on selective solid MM medium supplemented with 10 or 20 μg/mL hygromycin B (Sigma-Aldrich). All solid cultures were incubated at 30°C.

Liquid cultures were carried out in 250-mL flasks containing 100 mL liquid medium MM1 or MM2. MM1 contained 76 mM (NH_4_)_2_SO_4_ (Sigma-Aldrich), 1 mM CaCl_2_, 11 mM KH_2_PO_4_, 2 mM MgSO_4_, 50 g/L D-glucose and trace elements. In MM2, 151.4 mM of NaNO_3_ was used as nitrogen source instead of (NH_4_)_2_SO_4_ in order to minimize the impact of (NH_4_)_2_SO_4_ consumption on pH acidification. Different carbon sources were used in MM2, at 50 g/L: D-glucose, D-xylose (Sigma-Aldrich), L-arabinose (Sigma-Aldrich), potato starch (Fluka, Sigma-Aldrich) and birchwood xylan (Sigma-Aldrich). When stated, an extra 20 g/L glucose was added to the medium at 96 h of culture. For experiments with added CaCO_3_, 8 g of sterilized CaCO_3_ was added to the cultures after 24 h.

Liquid culture media were inoculated with 10^6^ spores/mL. To avoid germination problems in MM2 liquid medium containing pentose monomers and complex substrates, culture media were inoculated using 1 mL of mycelium from a 24 h preculture in Sabouraud broth.

Liquid cultures were incubated at 30°C under shaking at 140 rpm (50 mm orbital).

### Expression vector construction and fungal transformation

The *ldhA* (lactate dehydrogenase A gene) sequence of *R. oryzae* (Genbank accession number AF226154) was codon-optimized for expression in *Aspergillus* and synthesized by Life Technologies. The construct contained a *NcoI* and *HindIII* at the 5′ and 3′ ends, respectively, for cloning into pAN52.3 (kindly supplied by Pr. P. J. Punt, TNO, The Netherlands). The recombinant gene inserted into the expression vector was sequence-checked before transformation. Fungal co-transformation of *A. brasiliensis* BRFM103 was carried out as previously described [44] using pAN52.3 *ldhA* vector and pAN7.1 co-vector (kindly supplied by Pr. P. J. Punt, TNO, The Netherlands). The pAN7.1 co-vector contained the *hph* selection marker conferring hygromycin B resistance. Hygromycin B inhibition tests were carried out with the wild-type *A. brasiliensis* BRFM103 strain grown on MM solid medium supplemented with 10, 30, 50, 75, 100, 150, 200, 300, and 400 μg/mL hygromycin B (Sigma Aldrich) inoculated with spore suspensions. Growth inhibition tests showed that low hygromycin B concentration (10 μg/mL) slowed fungal growth and higher (above 10 μg/mL) hygromycin B concentrations prevented growth. Transformant screening was therefore done on MM solid medium supplemented with 20 μg/mL of hygromycin B.

### Organic acid production and sugar consumption

Samples were taken from the liquid screening medium during the incubation, and mycelia were removed by centrifugation. The pH values of the supernatants were determined with an Inolab pH l Level 1 electrode (WTW, Weilheim, Germany).

For transformant screening, L-LA was detected spectrometrically by measuring ABTS oxidation at 420 nm as previously described [45]. The reaction mix contained ABTS 0.5 mM, HRP (1.5 mM) and 1.5 mM lactate oxidase in 1 M phosphate buffer pH6.

The culture supernatants of L-LA-producing strains were analyzed by HPLC (Agilent 1100 series HPLC or Dionex Ultimate 3000 series, ThermoScientific) for organic acids and sugars using an Aminex HPX-87H Organic Acid Analysis Column (100 mm × 7.8 mm, Biorad, Marne-la-Coquette, France). The column was equilibrated in 2.5 mM H_2_SO_4_ in water at 35 or 30°C, and samples were eluted with 2.5 mM H_2_SO_4_ at a 0.6 mL/min flow rate. Sugars and ethanol were detected with a differential refractometer (HP1047A Hewlett Packard or Dionex Ultimate 3000 RI detector, Thermoscientific). Organic acids were detected with a UV detector (Agilent 1100 series wavelength detector or Dionex Ultimate 3000 series RS variable wavelength detector, ThermoScientific). Data were acquired using ChemStation (Agilent, Hewlett Packard) or Chromeleon (Dionex, Thermoscientific) software.

### Dry biomass determination

Dry biomass was determined by gravimetric method. At the end of the culture, the biomass was collected onto nylon filters (Miracloth, pore size of 22–25 μm) and washed with 200 mL distilled water to remove residual media. Washed biomass was then oven-dried at 105°C until a constant weight was reached (approximately 48 h).

### Determination of *ldhA* gene copy numbers and intracellular LDH activity

*ldhA* gene copy numbers in *A. brasiliensis*-*ldhA* strains were determined by quantitative real-time PCR (qPCR). Mycelia were harvested at 24 h of culture onto a nylon filter and washed with 100 mL distilled water. For each strain, 100 mg of mycelium was ground down (Fast prep-24®, MP Biomedicals), and genomic DNA was extracted using a Nucleospin Plant II kit (Macherey-Nagel, Germany). Concentration and purity of genomic DNA were determined by UV detection using a Nanodrop® spectrometer (Nanodrop2000c, Thermoscientific). Genomic DNA was considered pure enough when A_260/280_ and A_260/230_ were above 1.8 and 1.6, respectively. Monocopy Translation Elongation Factor 1α (*tef1*α) and RNA Binding Protein 1 (*rbp1*) genes were selected as internal calibrator and internal control, respectively. Specific primers for *ldhA* (forward: 5′-GATAAGACCGCCATCTCCAA-3′; reverse: 5′-CTTCTCGACGTAGACGG-3′), *ef1*α (forward 5′-AGGTCATCGTCCTCAACCAC-3′; reverse 5′-TGGGGGAAGATTCAACAGAC-3′) and *rbp1* (forward: 5′-CCGAAGGCTATGCAGAAGTC-3′; reverse: 5′-AGACAAGTTGGGGTCACCAG-3′) were designed using Primer3 [46]. Amplification efficiencies were determined using 4 serial dilutions of genomic DNA ranging from 1 to 1.10^−3^ ng/μL, and primers were validated. Each reaction mix comprised 2 μL genomic DNA (1 ng/μL), 0.3 μL forward and reverse primers (10 μM each) and 5 μL Sso AdvancedTM SYBR® Green Supermix (Bio-Rad, United States). The qPCR reactions were performed in technical duplicates on a CFX96 Touch Real-Time PCR Detection System (Bio-Rad) at 95°C for 30 sec, then 40 cycles at 95°C for 5 sec and 58°C for 5 sec. Melting curve analysis was performed after the PCR to verify the Tm of the amplicons. Gene copy numbers of *ldhA* and *rbp1* were calculated relative to *tef1*α by the ΔCt method using the amplification efficiency of each analyzed gene. *rbp1* gene copy number needs to be 1 in order to validate the qPCR run.

For intracellular LDH activity, mycelia were harvested at 72 h of culture onto a nylon filter and washed with 100 mL of MOPS buffer pH 7.2. The mycelia were stored at −80°C until grinding in liquid nitrogen using a Freezer Mill® 6770 (SPEX Sample Prep; Metuchen, NJ), then 200–300 mg of frozen mycelia powder was aliquoted into 2 mL tubes and suspended in extraction buffer (MOPS 1 M, 10% glycerol, 1 mM DTT, pH 7.2), 20% w/v. Samples were centrifuged at 14,500 *g* for 5 min at 4°C. NADH reductive activities in the supernatants were determined spectrophotometrically (A_340nm_) by measuring the kinetics of NADH,H^+^ reduction (Sigma-Aldrich, 175 μM) with and without pyruvate (Sigma-Aldrich, 40 mM) in 1 M MES buffer, pH 6.8. For each supernatant, the difference between NADH, H^+^ reductive activity with and without pyruvate was associated to combined LDH activity plus alcohol dehydrogenase (ADH) activity. Total protein concentrations were determined using the Bradford method. NADH reductive activities were expressed in Units (U) per mg of protein, where one U was defined as the amount of μmole NADH,H^+^ reduced by the LDH (or ADH) per minute. NADH reductive activities were measured in triplicate. Since the wild-type strain did not produce L-LA but ethanol, *in vitro* NADH consumption in this strain was attributed to the action of pyruvate decarboxylase and ADH. The LDH dehydrogenase activity of the transformants was therefore obtained by subtracting the NADH dehydrogenase activity of the wild-type strain from the total NADH dehydrogenase activity of each transformant.

## Abbreviations

ADH: Alcohol dehydrogenase
*hph*: Hygromycin B phosphotransferase gene
L-LA: L-lactic acid
LDH: Lactate dehydrogenase enzyme
MM: Minimum medium
*pdc*: Pyruvate decarboxylase gene
*ldhA*: Lactate dehydrogenase gene (from *R. oryzae*)
*tef1*α: Translation elongation factor 1α gene
*rpb1*: RNA polymerase II large subunit gene
